# Citric Acid: Properties, Microbial Production, and Applications in Industries

**DOI:** 10.3390/molecules29010022

**Published:** 2023-12-19

**Authors:** Ewelina Książek

**Affiliations:** Department of Agroenginieering and Quality Analysis, Faculty of Production Engineering, Wroclaw University of Economics and Business, Komandorska 118–120, 53-345 Wrocław, Poland; ewelina.ksiazek@ue.wroc.pl; Tel.: +48-697-377-695 or +48-71-36-80-289

**Keywords:** citric acid, food additives, *Aspergillus niger*, *Yarrowia lipolitica*, biosynthesis, citric acid market

## Abstract

Citric acid finds broad applications in various industrial sectors, such as the pharmaceutical, food, chemical, and cosmetic industries. The bioproduction of citric acid uses various microorganisms, but the most commonly employed ones are filamentous fungi such as *Aspergillus niger* and yeast *Yarrowia lipolytica*. This article presents a literature review on the properties of citric acid, the microorganisms and substrates used, different fermentation techniques, its industrial utilization, and the global citric acid market. This review emphasizes that there is still much to explore, both in terms of production process techniques and emerging new applications of citric acid.

## 1. Introduction

Citric acid (CA), also known as 2-hydroxypropane-1,2,3-tricarboxylic acid, is found in plant and animal tissues such as blood, bone, and muscle. For living organisms, citric acid is one of the essential carboxylic acids in the Krebs cycle, a series of reactions that oxidize glucose into carbon dioxide and water, releasing energy. Due to its harmless nature and chelating and sequestering properties for metal ions, citric acid has applications in the food, pharmaceutical, chemical, and even metallurgical industries [1,2]. The annual global production of citric acid currently reaches approximately 2.8 million tons, and the citric acid market is one of the fastest-growing segments in the food additive industry [3]. The continuous growth in citric acid production is attributed to its wide-ranging applications, not only in the food and pharmaceutical industries but also in biopolymer production, environmental protection, and biomedicine [4,5].

In industrial citric acid production, the dominant method is submerged fermentation involving strains of *Aspergillus niger*, yeast *Yarrowia lipolytica*, and some bacterial strains [6]. *Aspergillus niger* is considered the best among microorganisms in the commercial synthesis of citric acid due to its high production efficiency [7,8]. The development of citric acid production has significantly increased since the last century, thanks to biotechnology, which provides knowledge about fermentation techniques and product recovery; biochemistry, which provides insights into various factors influencing citric acid synthesis and inhibition; and molecular regulatory mechanisms and strategies to enhance citric acid production efficiency [1,9].

In this review, an attempt has been made to gather and update data on the biosynthesis of citric acid by the filamentous fungus *Aspergillus niger* while also highlighting the differences between it and the yeast *Yarrowia lipolytica*. The review addresses the progress in citric acid bioproduction, optimal fermentation strategies, and the utilization of conventional and unconventional carbon sources. Additionally, it discusses the prospects and future trends of the global citric acid market.

## 2. Physical and Chemical Properties

**Figure 1:**
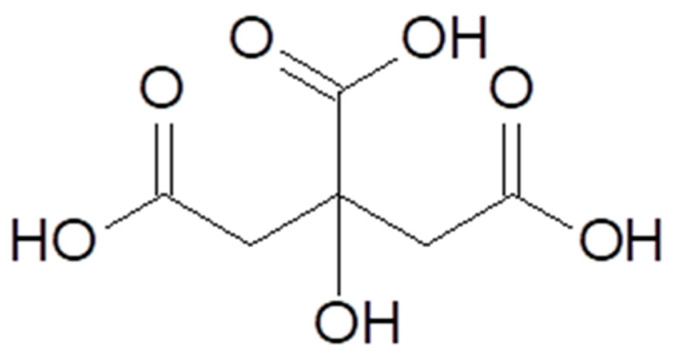
Chemical structure of citric acid.

Citric acid [77–92–2], according to IUPAC nomenclature (International Union of Pure and Applied Chemistry), is also known as 2-hydroxypropane-1,2,3-tricarboxylic acid. Citric acid is a polyprotic α-hydroxy acid but can also be classified as a *β*-hydroxy acid (Figure 1) [8,10]. It is present in plants, animal cells, and physiological fluids. In small quantities, citric acid is found in citrus fruits, especially lemons and limes. In amounts exceeding 1% of the dry weight of the product, it is present in lemons (4–8%), blackberries (1.5–3.0%), grapefruits (1.2–2.1%), as well as oranges, raspberries, and strawberries in the range of 0.6–1.3% [11,12,13].

Citric acid is an organic compound, a tricarboxylic hydroxy acid, with three carboxylic functional groups. It is a triprotic compound that undergoes three constant dissociations, which allows it to form three types of salts and exhibit buffering properties. The chemical and physical properties of citric acid are presented in Table 1 [14,15]. Citric acid forms crystalline mono-, di-, and tri-basic salts with various cations. From a technological perspective, the most important are calcium citrate, potassium citrate, and sodium citrate [16].

Citric acid is a weak acid in two crystalline forms: Anhydrous citric acid (C_6_H_8_O_7_) and monohydrated citric acid (C_6_H_8_O_7_·H_2_O). Anhydrous citric acid crystallizes from a hot concentrated solution above 36.6 °C, forming a white crystalline powder. On the other hand, monohydrated citric acid crystallizes from a cold solution at temperatures below 36.6 °C, forming colorless, transparent crystals [16,17]. Anhydrous citric acid absorbs a small amount of water at 25 °C and relative humidity in the 25 to 50% range. If the humidity is between 50% and 75%, it absorbs water significantly, while approaching 75% relative humidity takes the form of a monohydrate. The anhydrous form of citric acid is obtained when the relative humidity is less than 40%. Monohydrated citric acid slightly absorbs moisture at a relative humidity of 65–75% [17].

Citric acid is highly soluble in water and organic solvents such as ethanol, 2-propanol, ether, ethyl acetate, 1.4-dioxane, tetrahydrofuran, acetonitrile, and ethanol-water mixtures [18]. It has a higher solubility in alcohol than in water. Adding alcohol to an aqueous solution significantly increases the solubility of citric acid [19,20]. The solubility of citric acid in different solvents can be ranked as follows: Tetrahydrofuran < 1.4-dioxane < water < 2-propanol < ethanol < acetonitrile [21]. Citric acid does not dissolve in chloroform, toluene, benzene, carbon disulfide, or tetrachloride [17]. Its solubility increases with an increasing temperature of 20.55–60.05 °C [19,20,21].

When heated to 150 °C, citric acid remains stable, losing only its crystalline water. Above 175 °C, it undergoes a melting and decomposition process. Dehydration of citric acid leads to the formation of trans-aconitic acid. It is assumed that further thermal transformations of trans-aconitic acid due to dehydration result in the production of aconitic anhydride or a mixture of both isomers [15,22].

Citric acid can chelate metal ions by forming bonds between the metal, carboxyl, and hydroxyl groups of the citric acid molecule. Citric acid and its salts form complexes with copper, nickel, iron, magnesium, zinc, and tin. This valuable property helps prevent changes in chemical potential, precipitation of solids, or changes in chemical properties [15,23].

Citric acid esterifies with alcohols under typical conditions in the presence of catalysts such as sulfuric acid, *p*-toluenesulfonic acid, or ion-exchange resin. The esterification reaction of citric acid with alcohols, occurring at temperatures above 150 °C, does not require the presence of a catalyst. Citric acid forms polyesters with polyalcohols such as sorbitol and mannitol. Interrupting the esterification reaction before completion results in the formation of free carboxylic groups, forming salts [15].

**Table 1:** Chemical and physical properties of citric acid.

Properties	Characteristic	References
Molar mass	Anhydrous: 192.12 g∙mol^−1^ Monohydrate: 210.14 g∙mol^−1^	[11]
Appearance and form	powdery, colorless transparent crystals or white, granular, fine powder Anhydrous: monoclinic holohedral crystals. Monohydrate: rhombic crystals	[14]
Melting point	Anhydrous: 153° C Monohydrate: ≈100 °C	[8]
Boiling point	None, decomposition into water and CO_2_ > 175 °C	[24]
Vapor pressure	1.7 × 10^−8^ mmHg at 25 °C	[18]
Density	Anhydrous: 1.665 g∙cm^−3^ at 20 °C Monohydrate: 1.542 g∙cm^−3^ at 20 °C	[14]
Octanol/water partition coefficient	log_POW_ = −1.72 ± 0.4 at 20 °C	[10]
Partition coefficient	log_P_ = −1.198 ± 0.4 w 25 °C	[14]
dissociation constant	pK_1_ = 3.128 pK_2_ = 4.761 at 25 °C pK_3_ = 6.396	[14]
Henry’s constant	K_H_ = 2.3 ×·10^−7^ Pam^3^∙mol^−1^	[25]
Solubility	In water, the solubility increases with temperature: solubility is 54% at 10 °C. solubility is 84% at 100 °C. In alcohol, the solubility is 59.1 g per 100 milliliters (g/100 mL) at 25 °C In a mixture of ether and alcohol, the solubility is 162 g per 100 milliliters (g/100 mL) at 25 °C	[14]

## 3. Citric Acid Biosynthesis

### 3.1. The Beginning of Citric Acid Production

**Figure 2:**
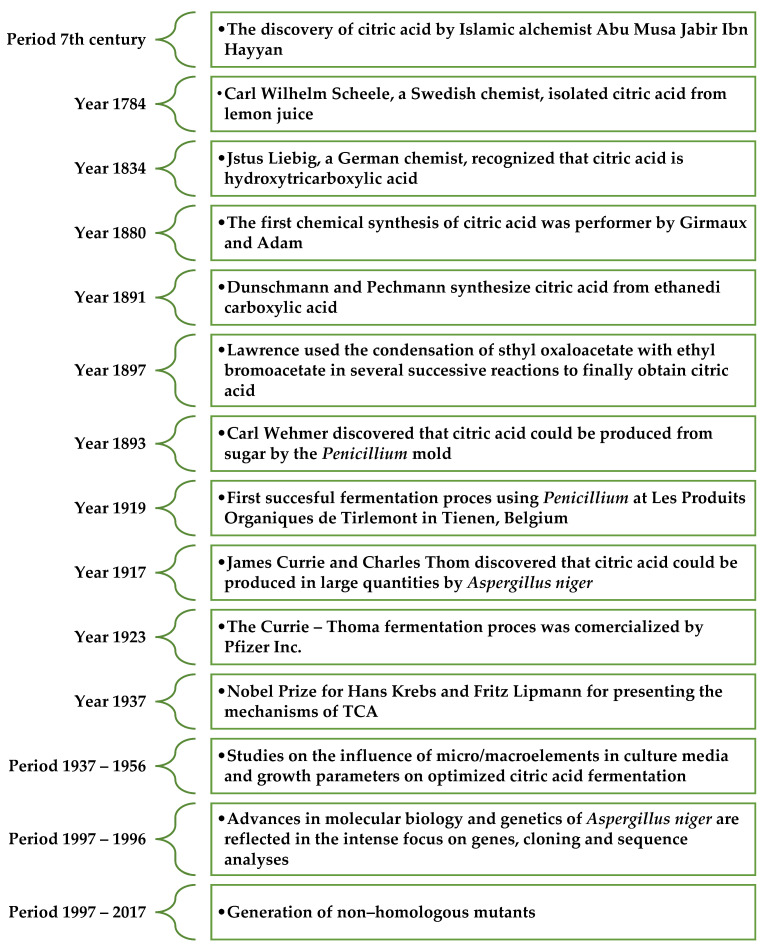
Major milestones in the discovery and research of citric acid [28,31].

For the first time, citric acid was isolated from lemon juice in 1784 in England by Carl Scheele, who obtained calcium citrate by adding lime to lemon juice [26]. In 1838, Liebig confirmed the presence of one hydroxyl group and three carboxyl groups in the structure of citric acid. Since 1860, citric acid production from lemons has been carried out in the United Kingdom, France, and Germany. Intensive research was conducted to find an alternative method for obtaining citric acid [27]. In 1893, the German botanist Wehemer observed that citric acid is formed as a byproduct during the production of calcium oxalates by Penicillium glaucum [26]. Industrial-scale production of citric acid involving microorganisms was initiated in 1917 by Currie, who developed a method for obtaining it from filamentous fungi *Sterigmatocystis nigra* (currently *Aspergillus niger*) using culture media containing sucrose [28,29]. The significant milestones in the discovery and research of citric acid are shown in Figure 2.

The biochemical foundations of the biosynthesis process of citric acid were elucidated in the 1950s with the discovery of glycolysis and the tricarboxylic acid cycle [30].

### 3.2. Microorganisms Producing Citric Acid

Since discovering the potential for microorganisms to produce citric acid, *Aspergillus niger* strains have remained the preferred microorganisms in the production process [26,29,32,33,34,35]. In addition to filamentous fungi such as Aspergillus niger, various other microorganisms have been used to produce citric acid. These include *Aspergillus nidulans*, *Aspergillus aculeatus*, *Aspergillus fumaricus*, *Aspergillus carbonarius*, *Aspergillus awamori*, *Aspergillus wentii*, *Aspergillus saitoi*, *Aspergillus flavus*, *Aspergillus foetidus*, *Aspergillus fonsecaeus*, *Aspergillus luchensis*, *Aspergillus phoenicis*, *Aspergillus saitoi*, *Aspergillus usumii*, *Penicillium janthinellum*, *Penicillium restrictum*, *Trichoderma viride*, *Mucor piriformis*, *Talaromyces* sp., *Eupenicillium* sp., *Botrytis* sp., *Absidia* sp., and *Ustulina vulgaris*. Additionally, promising producers of citric acid include yeasts such as *Yarrowia lipolytica*, *Candida tropicalis*, *Candida guilliermondii*, *Candida intermedia*, *Candida parapsilosis*, *Candida zeylanoides*, *Candida fibriae*, *Candida subtropicalix*, *Candida oleophila*, and bacteria such as *Arthrobacter paraffineus*, *Bacillus licheniformis*, and *Corynebacterium* spp. These microorganisms are all potential sources for citric acid production [36,37,38,39].

#### 3.2.1. Production of Citric Acid Using *Aspergillus niger* Fungi

**Figure 3:**
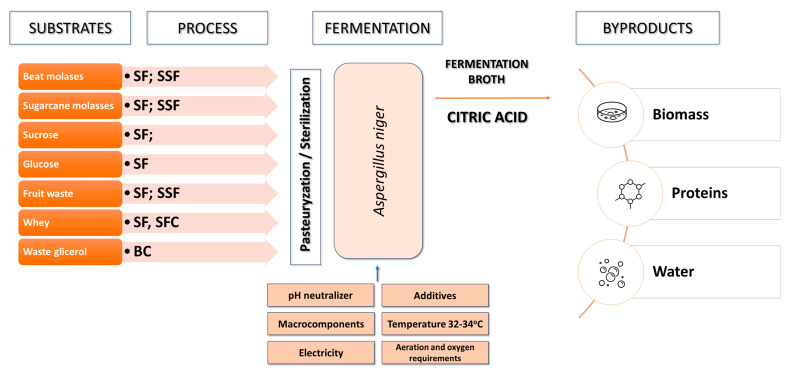
Citric acid production processes with *A. niger*.

In industrial citric acid production, filamentous fungi, mainly *Aspergillus niger*, are used (Figure 3) [40,41]. These microorganisms offer several advantages, including their ability to quickly adapt and grow on various substrates, regulate and control metabolic pathways, and regulate the secretion of citric acid from both mitochondria and cytosol. This contributes to citric acid accumulation and prevents its degradation in the Krebs cycle. Moreover, cultures using *Aspergillus niger* are characterized by high production efficiency and homofermentative citric acid biosynthesis [32,42]. *Aspergillus niger* strains have been recognized as safe, as they do not produce ochratoxin under controlled cultivation conditions and do not elicit strong allergic reactions in humans. In addition to citric acid and other organic acid biosynthesis, *Aspergillus niger* is also utilized to produce enzymes such as pectinases, proteases, aminoglycosidases, catalases, lipases, and oxidases [43].

Until 1980, *Aspergillus niger* strains used in industrial production were obtained through screening and mutagenesis. Mutagenesis techniques are still in use and continue to yield positive results in improving biosynthesis efficiency. The most commonly used mutagens are physical factors (gamma and UV radiation), chemical factors, and hybrid methods that combine physical and chemical characteristics [44]. The development of genetic engineering has allowed the application of DNA recombination technologies to improve strains, with *Aspergillus niger* being used as a host for the expression of heterologous proteins. The improvement of *Aspergillus niger* strains is limited by the relatively small number of available plasmid vectors for these filamentous fungi. Recently, integration and autonomous replication of plasmid vectors have been used to manipulate the genome with targeted gene exchange [1,2,45,46].

In order to enhance the performance of species such as *Aspergillus niger,* research has been conducted on the application of genome editing technology in filamentous fungi, employing CRISPR (Clustered Regularly Interspaced Palindromic Repeats) elements with associated endonucleases (such as Cas protein family members). The CRISPR/Cas9 system facilitates chromosome engineering in *Aspergillus niger*, enabling genome manipulations with high efficiency and scalability, thereby increasing the implementation speed of metabolic engineering cycles [46,47]. In experiments involving the production of natural metabolites, genome editing disrupted pyrG, which encodes orotidine-5′-decarboxylase, resulting in a 2.17-fold increase in citric acid production compared to the control, suggesting that inhibiting uridine/pyrimidine synthesis may promote citric acid overproduction [48].

#### 3.2.2. Citric Acid Metabolism in *Aspergillus niger*

The biochemical mechanism through which *Aspergillus niger* accumulates citric acid has been the subject of scientific interest since the 1930s, when cultivation conditions were established and the impact of various substrate components was assessed for industrial production needs. Despite numerous models proposed since 1930 to explain *Aspergillus niger’s* ability to accumulate citric acid, many aspects of the biochemical transformations remain unexplained. Research aimed at understanding the metabolic pathways and properties of enzymes in *Aspergillus niger* has made it the best-studied filamentous fungus [49].

The ability of certain strains of *Aspergillus niger* to biosynthesize citric acid is determined genotypically while providing appropriate cultivation conditions, and their control allows for achieving high process yields [50].

**Figure 4:**
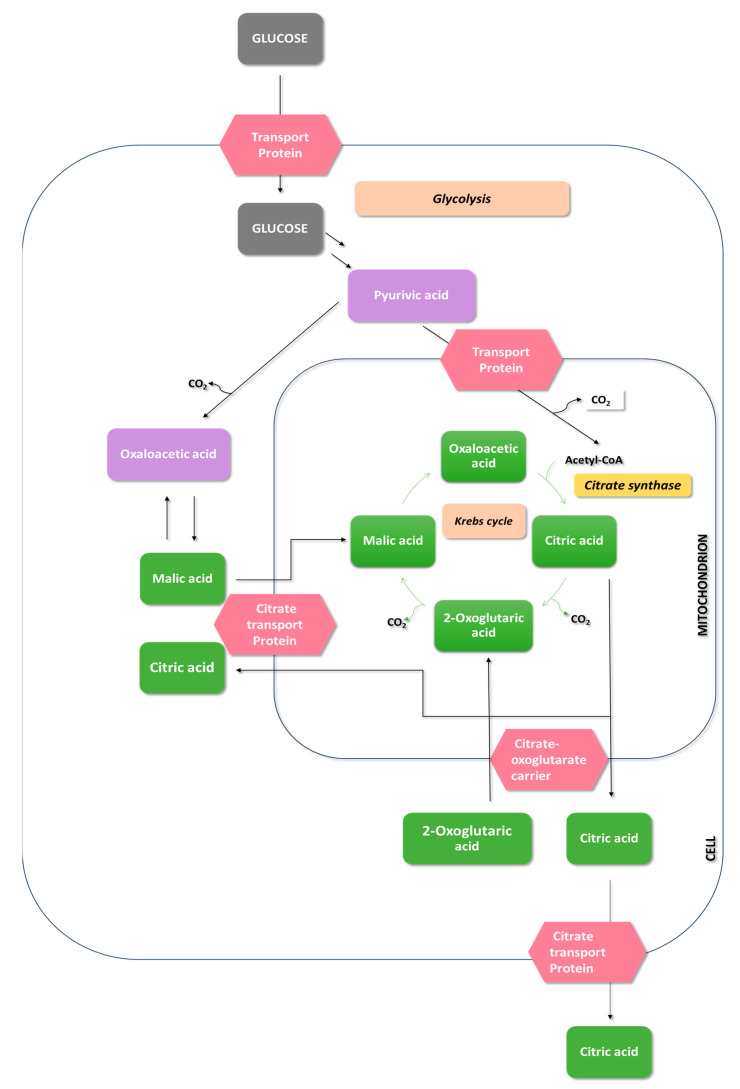
Overview of pathways leading to citric acid production in *A. niger*.

In the trophophase, during the growth of *Aspergillus niger* mycelium, hexoses or other carbohydrates are taken up through glycolysis and the pentose phosphate pathway (Figure 4). The involvement of the pentose phosphate pathway in carbohydrate metabolism is shallow and significantly decreases during citric acid production—the idiophase [51].

Glucose, upon entering the cell, undergoes phosphorylation, converting into glucose-6-phosphate. This step is catalyzed by hexokinase and glucokinase. Hexokinase is an enzyme that catalyzes the transfer of phosphate groups from ATP to glucose and fructose. On the other hand, glucokinase exhibits high affinity only for glucose [52]. The activity of hexokinase is strongly inhibited by trehalose-6-phosphate. To increase the efficiency of citric acid biosynthesis, the gene tpsA, responsible for the expression of trehalose-6-phosphate synthase, was blocked [53,54].

The next step in glycolysis is the isomerization of glucose-6-phosphate to fructose-6-phosphate. This reaction is catalyzed by phosphoglucose isomerase. Following the isomerization reaction, a phosphorylation reaction catalyzed by phosphofructokinase, a key enzyme in the glycolytic pathway, occurs. As a result of this reaction, fructose-6-phosphate is transformed into fructose-1,6-bisphosphate [55]. Phosphofructokinase activity is inhibited by a high concentration of ATP, leading to a decrease in its affinity for fructose-6-phosphate. This means that the activity of phosphofructokinase increases when the energy charge decreases [56]. Inhibitors of phosphofructokinase also include a high concentration of manganese, citrate, and phosphoenolpyruvate. Stimulating effects on phosphofructokinase activity are exerted by NH^4+^, Zn^2+^, Mg^2+^, and adenosine monophosphate (AMP) [49,57].

The first reaction in the third stage of glycolysis is the conversion of 3-phosphoglyceraldehyde to 1,3-bisphosphoglycerate. This process is catalyzed by 3-phosphoglyceraldehyde dehydrogenase and involves the oxidation of the aldehyde with the participation of NAD^+^ to form a carboxylic acid and the production of 1,3-bisphosphoglycerate. In the next stage of glycolysis, phosphoglycerate kinase catalyzes the transfer of a phosphate group from 1,3-bisphosphoglycerate, forming ATP and 3-phosphoglycerate. The final stage of glycolysis involves the conversion of 3-phosphoglycerate to pyruvate, accompanied by the production of ATP. In this stage, the critical enzyme is pyruvate kinase, which catalyzes the irreversible transfer of a phosphate group to ATP [58,59].

In the glycolytic pathway, glucose is converted into two molecules of pyruvate. Pyruvate transforms precursors such as citrate, oxaloacetate, and acetyl-CoA. Oxaloacetate is formed through the carboxylation of pyruvate, catalyzed by pyruvate carboxylase. During this reaction, *Aspergillus niger* consumes CO_2_ generated during acetyl-CoA formation [50]. Pyruvate is then transported to the mitochondrion, where it undergoes oxidative decarboxylation to form acetyl-CoA in an irreversible reaction catalyzed by pyruvate dehydrogenase. The reaction of acetyl-CoA formation from pyruvate serves as a bridge between the glycolytic pathway and the citric acid cycle. The citric acid cycle occurs in the mitochondrial matrix and begins with oxaloacetate, acetyl-CoA, and H_2_O condensation to form citrate and CoA. The enzyme catalyzing this reaction is citrate synthase. Subsequently, citrates undergo isomerization to isocitrate through reactions catalyzed by aconitase [13,60,61].

In the past, it was speculated that the accumulation of citric acid in the citric acid cycle occurs due to the inhibition of aconitase, isocitrate dehydrogenase-NADP, and α-ketoglutarate dehydrogenase by external factors (metal ions, pH, Cu^2+^) or internal factors (glycerol, citrates) [49,52].

The accumulation of citric acid is associated with the activity of tricarboxylate transporters that compete with aconitase for citrates. The affinity of the tricarboxylate transporter for citrate is significantly higher than that of aconitase. Consequently, citrates are released from the mitochondrion without inhibiting the Krebs cycle. The transport of citrates by tricarboxylate carriers operates through exchange with cytosolic malate. Hence, malate can be considered a potential trigger for the accumulation of citric acid, as an increase in its concentration precedes the accumulation of citrates [49].

#### 3.2.3. Production of Citric Acid Using *Yarrowia lipolytica* Yeast

**Figure 5:**
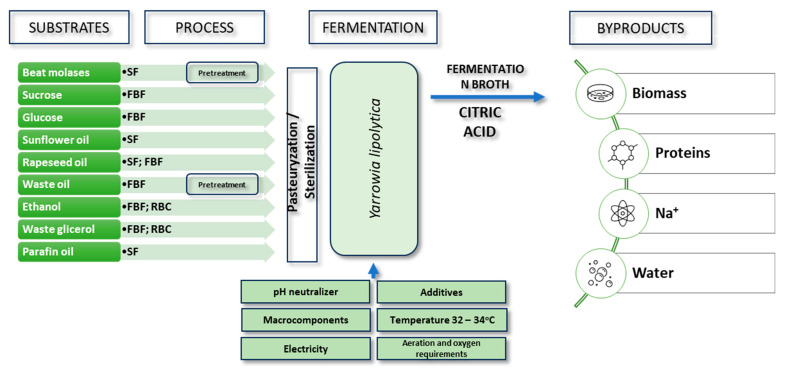
Citric acid production processes with *Y. lipolytica*.

Yeast, especially *Yarrowia lipolytica* and *Candida* strains, have been used in citric acid production since the 1960s [62]. Initially, n-alkanes were used as carbon sources in cultures, but over time, other substrates such as glucose, acetates, molasses, glycerol, inulin, oils, and fatty acids were introduced (Table 2; Figure 5) [63,64,65].

**Table 2:** Substrates used in citric acid production by *Yarrowia lipolytica* strains.

Substrate	Strain	Citric Acid	Cultivation Method	References
Glucose	*Yarrowia lipolytica*	121–129 g∙dm^−3^	SF	[66]
	*Yarrowia lipolytica*	49 g∙dm^−3^	SF	[67]
	*Yarrowia lipolytica VKM Y-2373*	80–85 g∙dm^−3^	FBF	[68]
	*Yarrowia lipolytica NRRL Y-1094*	30.31 g∙dm^−3^	SF	[69]
Pure glycerol	*Yarrowia lipolytica* NG40/UV7	115 g∙dm^−3^	SF	[70]
Waste glycerol	*Yarrowia lipolytica* NG40/UV7	112 g∙dm^−3^	SF	[70]
	*Yarrowia lipolytica* SKY7	18.70 g∙dm^−3^	FBF	[38]
	*Yarrowia lipolytica* A101	75.9 g∙dm^−3^	SF	[71]
Inulin	*Yarrowia lipolytica* SWJ-1b	85 g∙dm^−3^	SF	[72]
	*Yarrowia lipolytica AWG7 INU 8*	200 g∙dm^−3^	RBF	[73]
Waste cooking oil	*Yarrowia lipolytica* SWJ-1b	31 g∙dm^−3^	SF	[74]

The main advantages of *Yarrowia lipolytica* strains compared to filamentous fungi include better tolerance to high carbon source concentrations, lower sensitivity to heavy metal ions, and lower oxygen levels in the growth medium. This allows for the utilization of a wide range of substrates. Moreover, citric acid biosynthesis using *Yarrowia lipolytica* yeast is characterized by higher efficiency, faster production, and easier control [40,75,76,77].

**Figure 6:**
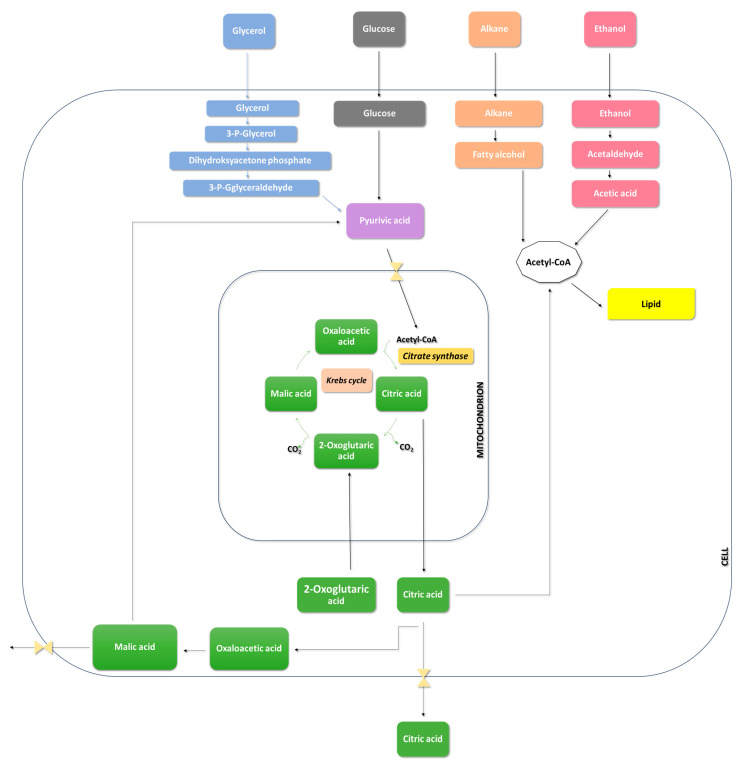
Metabolic pathways of *Y. lipolytica*.

The accumulation of citric acid in yeast requires a deficiency in a nitrogen source because its production initiates after the depletion of available nitrogen. Citrate synthase, the enzyme converting oxaloacetate and acetyl-CoA into citric acid, is modulated by the provision of ammonium ions in the medium. Therefore, ensuring a high C/N ratio is crucial so that the excess carbon is redirected towards citric acid production in the stationary growth phase (Figure 6) [78,79].

Yeast also offers the advantage of the ease of genetic modifications using molecular techniques and eliminates the need for prior substrate processing [42]. Tan et al. expressed the pyruvate carboxylase gene cloned from *Meyerozyma guilliermondii* in *Yarrowia lipolytica* SWJ-1b to enhance citric acid production. The research resulted in both increased pyruvate carboxylase activity and citric acid production by the obtained recombinant *Yarrowia lipolytica* [63]. On the other hand, Liu et al. increased the expression of the ICL1 gene and reduced the ACL1 gene of the ATP citrate lyase to enhance citric acid production by *Yarrowia lipolytica.* The result of these studies was citric acid production reaching 84.0 g∙dm^−3^ within 214 h [80].

A significant challenge in citric acid production with yeast is the concurrent secretion of isocitric acid, which is undesirable and interferes with crystallization [80]. The amount of accumulated citric acid depends on the yeast strain and carbon source used. In culture media containing vegetable oils or n-alkanes as the carbon source, the proportion of isocitric acid is around 35–45%, while in glycerol-based media, it is about 10–12% [81]. To reduce the presence of isocitric acid in the culture medium, strains have been improved using genetic engineering methods, such as inducing overexpression of isocitrate lyase, resulting in a significant reduction in isocitrate levels, or increasing the activity of pyruvate carboxylase [63,75].

The issue of citric acid production by yeast has been extensively described in the work by Börekçi et al., “Citric Acid Production of Yeasts: An Overview” [69].

## 4. Production of Citric Acid

### 4.1. Cultivation Methods and Conditions

Currently, over 90% of the world’s citric acid production is manufactured using three methods: Submerged fermentation (SF), liquid surface fermentation (LSF), and solid-state fermentation (SSF) [37]. The advantages and disadvantages of different cultivation methods used in citric acid biosynthesis are presented in Table 3.

**Table 3:** Advantages and disadvantages of different cultivation methods used in the biosynthesis of citric acid by *Aspergillus niger*.

Type of Cultivation	Process Parameters	Process Advantages	Process Disadvantages	References
Surface cultivation in liquid substrates (LSF)	Process Duration: 8–12 days Process yield: 70–75%	Ease of operation Energy-efficient Technically simple	Long duration Sensitive to contamination by other microorganisms Requires large production areas Generates large amounts of heat Production on a small and medium industrial scale	[29,32,82]
Surface cultivation in solid substrates (SSF)	Process Duration: 4 days	Technically and technologically simple Low substrate cost Energy-efficient Low risk of contamination Low waste generation Low sensitivity to heavy metal pollution	Difficulties in controlling process parameters (pH, humidity, temperature) High product contamination High cost of product acquisition	[36,83,84,85]
Submerged fermentation cultures (SF)	Process Duration: 4 days	Ability to control process parameters High process efficiency Low production costs Ease of maintaining sterile conditions	Sensitivity to the inhibitory effects of trace elements A large amount of waste is generated 80% of the world’s citric acid production	[29,50,82]

#### 4.1.1. Liquid Surface Fermentation Cultures

**Figure 7:**
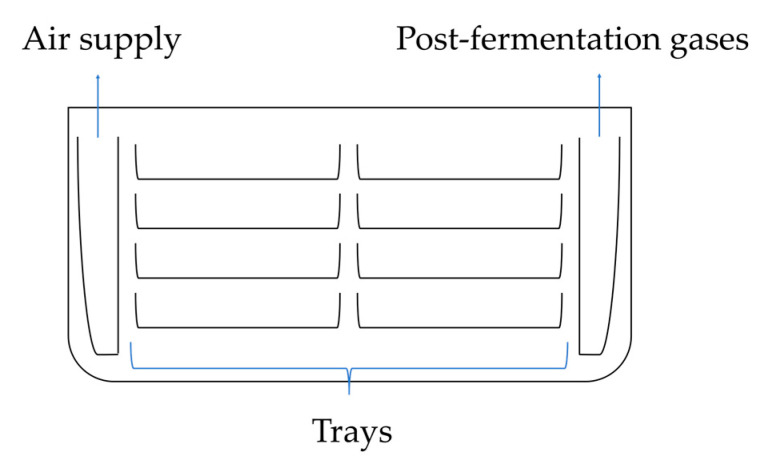
Schematic of liquid surface fermentation cultures.

This method is still used on a small and medium industrial scale due to its simple technology and low production costs [26]. In surface fermentation cultures, *Aspergillus niger* fungi grow on the surface of the growth medium and form a thick mycelial layer. This process occurs in fermentation chambers on high-quality steel, aluminum, or polyethylene trays (Figure 7). The fermentation chambers are equipped with an aeration system that controls temperature and humidity levels. The air supplied to the fermentation chambers is filtered using bacteriological filters to prevent contamination by *Penicillium*, other strains of *Aspergillus niger*, or lactic acid bacteria [4,86].

Surface cultivation generates significant heat, requiring a high aeration rate to maintain the proper temperature. This process generates significant heat during fermentation, which is controlled by proper aeration. The chamber requires adequate ventilation, and fermentation chambers have ensured efficient air circulation passing over the substrate’s surface through a bacteriological filter to control humidity and temperature through cooling. Carbon dioxide produced during the fermentation process inhibits the production of citric acid at concentrations higher than 10% [4,5].

#### 4.1.2. Solid-State Fermentation Cultures

This cultivation method involves the growth of microorganisms on solid substrates. Initially, the appropriate moisture is provided in the form of humidity in the raw material, and additional moisture is supplied by the air during the process. Solid-state cultivation is considered a reaction in a heterogeneous system with simultaneous multicomponent mass and heat transport [9,83].

**Figure 8:**
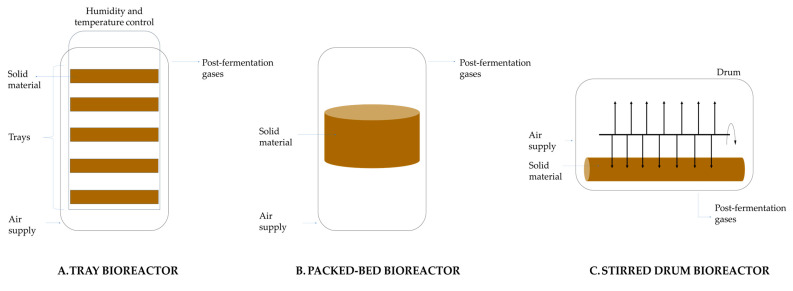
Schematic of solid-state fermentation cultures.

On a laboratory scale, SSF devices consist of media such as petri dishes or flasks in which screening tests can be performed. On an industrial scale, various types of bioreactors are used, which differ mainly in the presence or absence of mixing and forced aeration. The simplest type is a shelf bioreactor, in which solid material is placed on trays made of metal or plastic. The trays are placed in a chamber where circulating air regulates temperature and humidity. The second type of culture can take place in packed-bed column bioreactors. The third type is stirred drum bioreactors, which are used in SSFs requiring slow, continuous mixing and no forced aeration (Figure 8) [87,88].

This method can use waste from agriculture and industry, such as fruit and vegetable processing waste, as substrates. Drum, column, and rotary bioreactors are used for citric acid production on solid-state substrates [89].

One clear advantage of this method is its low energy consumption and minimal waste generation, which is environmentally friendly. Additionally, the process takes about four days under optimal conditions, significantly shorter than submerged and liquid surface fermentation cultures [32,90,91].

Solid-state cultures of *Aspergillus niger* have gained importance in recent years. Still, due to the low level of process automation and a need for improvements in bioreactor design, they are only marginally used in industrial citric acid production [92].

#### 4.1.3. Submerged Fermentation

**Figure 9:**
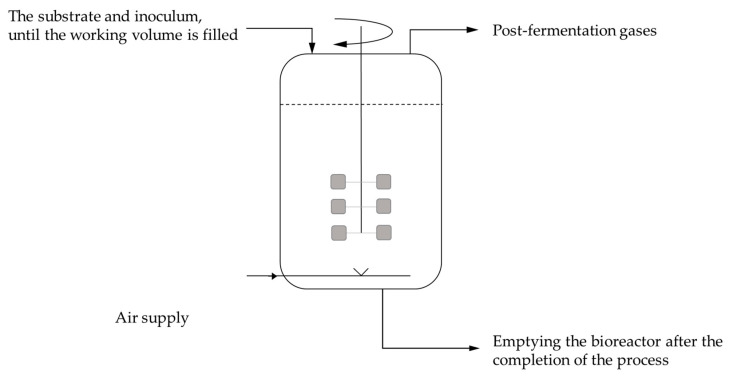
Schematic of submerged fermentation.

Around 80% of the world’s citric acid production is achieved using submerged fermentation. Citric acid production through batch culture is carried out in tank bioreactors of high-quality corrosion-resistant steel equipped with aeration and mixing systems (Figure 9). The most commonly used carbon source for citric acid production is sucrose, as well as by-products of its production, such as molasses [13,32].

The advantages of submerged culture over surface culture include lower costs, low contamination risk, a high level of automation, and higher process yield. To achieve high citric acid production yields in submerged cultures, control of process parameters and careful substrate selection are crucial [93].

**Figure 10:**
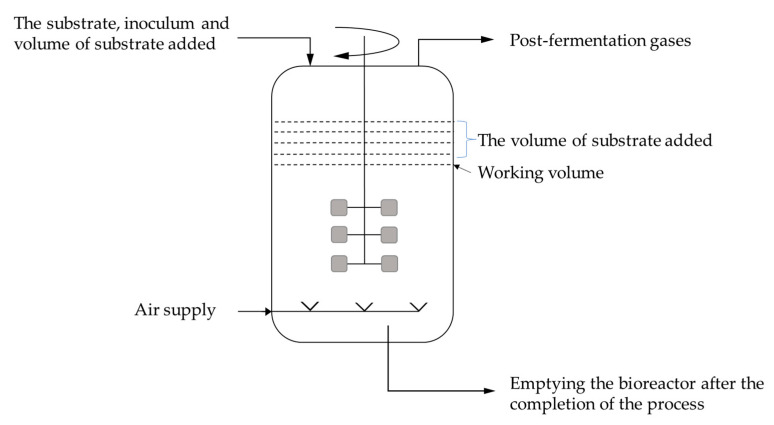
Schematic of fed-batch fermentation.

The periodic batch culture is the most frequently used method in industrial citric acid production. Other methods include fed-batch and semicontinuous cultures [94]. In fed-batch fermentation, sterilized nutrients are added to the fermenter during biomass growth (Figure 10). In continuous fermentation, sterilized liquid nutrients are introduced into the fermenter at the same flow rate as the fermenting wort leaving the system. Parameters such as temperature, pH, oxygen consumption, and carbon dioxide production are measured and controlled to optimize the fermentation process [95].

### 4.2. Factors Influencing Citric Acid Production

**Figure 11:**
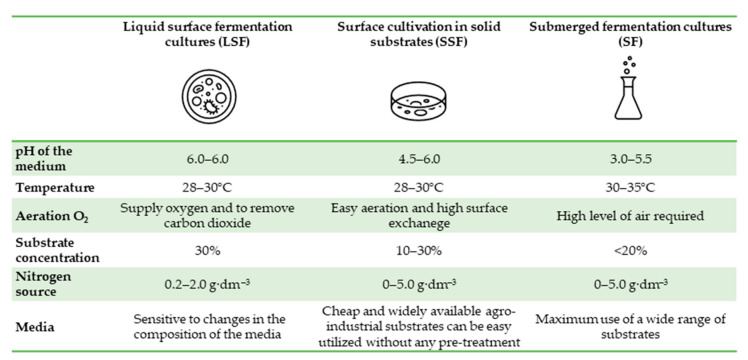
Summarizing the impact of factors that stimulate the citric acid biosynthesis process.

The course of *Aspergillus niger* cultivation and the rate of citric acid biosynthesis in submerged culture are influenced by many factors, including the type of carbon source and its concentration, the type and concentration of metal ions present in the culture media, low molecular weight alcohols, fungal morphology, as well as temperature, pH, aeration rate, and mixing rate. A brief summary of the impact of factors on citric acid biosynthesis in different cultivation methods is presented in Figure 11. The impact of factors that stimulate the citric acid biosynthesis process has been widely researched.

#### 4.2.1. Nitrogen

The concentration and source of nitrogen have a fundamental impact on the growth of *Aspergillus niger* and the biosynthesis of citric acid in both submerged and solid-state cultures. The most preferred nitrogen sources are nitrogen salts, including ammonium nitrate, ammonium sulfate, and ammonium chloride. Among other nitrogen sources, urea, peptone, and yeast extract can be distinguished [4]. According to the literature, ammonium nitrate is considered the most favorable nitrogen source [96,97,98,99,100].

Ammonium compounds lead to an advantageous reduction in the pH of the culture medium to a level lower than 2, which is a necessary condition for citric acid production. Limiting the nitrogen source during cultivation inhibits fungal biomass growth and increases citric acid production [50]. The optimal concentration of the nitrogen source should be around 0.2%, as it promotes the biosynthesis of citric acid by *Aspergillus niger.* The highest citric acid biosynthesis efficiency is achieved when the intracellular concentration of ammonium ions is 2–3 mM∙g^−1^. However, process efficiency decreases when the intracellular nitrogen ion concentration is 1 mM∙g^−1^ of biomass [97,98,99]. Culture media are supplied during cultivation to increase the volumetric citric acid biosynthesis rate. In addition to the proper amount of added nitrogen source, the timing of the addition and supplying it at the wrong fermentation phase can reduce the citric acid accumulation rate [26].

In the case of yeast, citric acid biosynthesis begins after the nitrogen source is depleted. Limiting the nitrogen concentration at a high substrate concentration for yeast is crucial because citric acid is released through a specific, energy-dependent transport system induced by intracellular nitrogen restriction [62].

#### 4.2.2. Phosphorus

The presence of phosphorus in the culture medium also influences the efficiency of citric acid biosynthesis. The best sources of phosphorus are KH_2_PO_4_ and K_2_HPO_4_. Limiting phosphorus concentration in the culture medium, similar to nitrogen, critically impacts citric acid production. Increasing the process efficiency is allowed by a phosphorus concentration in the range of 0.006 to 0.32 g∙dm^−3^. Fungal mycelium requires the presence of phosphorus at concentrations ranging from 0.01 to 0.02% for proper growth [90,101].

High phosphorus concentrations stimulate mycelial growth, induce secondary enzymatic reactions, and inhibit citric acid production [98].

#### 4.2.3. Trace Elements

Trace elements are a crucial factor affecting the efficiency of citric acid biosynthesis. Manganese, zinc, copper, and iron are of great importance [101]. To achieve high process efficiency, especially in submerged cultures, it is necessary to use culture media with controlled trace element content. This is due to their significant influence on the growth and physiology of *Aspergillus niger* and the efficiency of citric acid biosynthesis [50].

Magnesium is essential for the proper growth of *Aspergillus niger* and citric acid production due to its role as a cofactor in enzymatic reactions. Maximum citric acid biosynthesis efficiency is achieved at magnesium sulfate concentrations ranging from 0.020 to 0.025% [29,90].

The addition of manganese to the culture medium plays a significant role in the accumulation of citric acid, cell wall synthesis, sporulation, and the production of secondary metabolites [50]. At a concentration of 10 mg∙dm^−3^, manganese limits the efficiency of citric acid biosynthesis. However, at concentrations lower than 3 µg∙dm^−3^, it significantly reduces the process efficiency. Manganese deficiencies contribute to reduced lipid synthesis and increased cell membrane permeability due to decreased concentrations of certain enzymes involved in anabolic processes [102,103,104].

In citric acid production, limiting the presence of iron in the culture medium is crucial. Approximately 1 mg of iron per liter of culture medium is needed to achieve high process efficiency. Higher iron concentrations can lead to the accumulation of oxalic acid [90].

The presence of copper ions reduces the harmful effect of excess iron ions. Furthermore, copper is known to inhibit manganese ions. The optimal concentration of CuSO_4_∙7H_2_O in the culture medium should be 78 mg∙dm^−3^ [101]. The presence of copper at various concentrations affects the morphology of *Aspergillus niger* mycelium. Therefore, its presence determines the attainment of the appropriate mycelial structure, allowing for high efficiency in citric acid biosynthesis [50,105].

#### 4.2.4. Low-Molecular-Weight Alcohols

Potential stimulators of citric acid fermentation include low-molecular-weight alcohols, such as methanol, ethanol, and *n*-propyl alcohol [106,107]. Ethanol added to culture media inhibits mycelial growth and sporulation, reduces substrate consumption, increases cell membrane permeability, and influences an increase in citrate synthase activity and a reduction in aconitase activity [108,109,110].

Methyl alcohol, unlike ethanol, is not assimilated by *Aspergillus niger* and does not undergo conversion to acetyl-CoA, which is a precursor of the Krebs cycle. The mechanism of methanol’s interaction with citric acid biosynthesis in synthetic and natural media has not been fully elucidated. Methyl alcohol in high-purity media disrupts metabolic processes and biomass growth, reducing citric acid production. It also leads to disturbances in the synthesis of cellular proteins in the early stages of cultivation [111,112]. Methyl alcohol affects the permeability of cell membranes, which may be due to changes in the composition of phospholipids and triglycerides [113]. Phospholipids play a significant role in regulating membrane permeability for citric acid. Methyl alcohol may disrupt the formation of mycelial structure through chelation effects on metal ions such as copper (II), which play a significant role in regulating the content of fatty acids in glycolipids and phospholipids [114].

In the study by Maddox et al. [115], methanol in synthetic media containing galactose as a carbon source had a toxic effect by limiting fungal growth and reducing substrate consumption. However, it simultaneously increased the efficiency of citric acid production. Furthermore, methyl alcohol inhibited the activity of 2-oxoglutarate dehydrogenase, resulting in increased citric acid accumulation. Similarly, Yaykaşlı et al. [116] demonstrated that methanol in the biosynthesis process using immobilized conidia of *Aspergillus niger* in media with sucrose contributed to an increase in citric acid production.

Most of the available literature data report a positive impact of lower concentrations of methyl alcohol on the efficiency of citric acid production by *Aspergillus niger* in natural media characterized by lower purity, such as molasses-based media [106,111,113,117,118,119,120,121]. The stimulating effect of methyl alcohol added to natural media results from its limitation of the negative impact on the biosynthesis process of metal ions such as manganese, iron, and zinc, which strains of *Aspergillus niger* are highly sensitive to. Methyl alcohol, on the other hand, increases the tolerance of *Aspergillus niger* to the content of iron, manganese, and zinc ions present in the media [117]. Methyl alcohol induces changes in the normal carbohydrate metabolism pathway by increasing glycolytic capabilities, leading to citric acid accumulation. In natural media, it stimulates citric acid production by affecting fungal growth and altering the composition of the cell wall lipids [121].

#### 4.2.5. The pH Value

The pH value plays a significant role during spore germination, where the pH should be higher than 5, and during citric acid production, when a low substrate pH is required (pH ≤ 2). Most fungal mycelia grow in the pH range of 3 to 6, and their metabolic activity mainly depends on the culture medium’s pH [122,123,124].

During the production of citric acid by *Aspergillus niger* in submerged and surface cultures, a pH range of 2 to 6 is utilized. The low pH level reduces the risk of culture contamination by other microorganisms. Furthermore, a pH below 3 prevents the production of oxalic and gluconic acids [124]. Conversely, increasing the pH to 4.5 can lead to a significant reduction in citric acid production efficiency by up to 80% [50,125].

The pH of the culture medium can also affect the morphology of *Aspergillus niger mycelia*. At pH values of 2.0 to 2.2, the mycelia assume the desired form of small aggregates and short hyphae, which is most desirable for citric acid production. At a pH of 1.6, the morphological development of mycelia is disrupted, and process efficiency decreases significantly. In a medium with a pH of 3.0, mycelia form larger aggregates, favoring oxalic acid biosynthesis [50].

#### 4.2.6. Aeration and Mixing Rate

The appropriate dissolved oxygen concentration in the cultivation medium is a critical factor influencing the efficiency of citric acid biosynthesis [26,113]. The aeration rate significantly affects citric acid biosynthesis, especially in submerged cultivation methods. In submerged cultivations, the process efficiency increases with higher aeration rates and pressures (0.10–0.17 MPa) [90]. In cases of insufficient oxygen in the medium or interruptions in oxygen supply, citric acid production may be inhibited, and fungal growth may be affected [29,126,127]. The proper concentration of dissolved oxygen in the cultivation medium influences the rate of glycolysis and the respiratory chain, leading to high ATP levels and increased citrate secretion [57].

In industrial production, the aeration rate is adjusted according to the current fungal demand. Initially, the aeration rate is around 0.1 vvm, and during the phase of intensive fungal growth, it is increased to approximately 0.5 vvm [26]. To enhance the efficiency of citric acid biosynthesis in submerged cultivations, aeration rates ranging from 0.9 to 1.3 vvm have been applied [39]. Inappropriate aeration rates can have an adverse impact on the efficiency of the bioprocess [50].

Proper aeration, combined with high dissolved oxygen levels, contributes to the reduced activity of cytochrome-dependent enzymes and the increased activity of alternative oxidase, consequently favoring the alternative respiratory pathway. This process conserves energy by bypassing the need to generate ATP. As a result, citric acid fermentation requires continuous cooling because the free electron transport generates heat [49].

An essential parameter associated with aeration is the mixing rate, which affects fungal dispersion, dissolved oxygen concentration in the medium, and even enzyme activity, such as citrate synthase, aconitase, and isocitrate dehydrogenase. The activity of citrate synthase decreases with increasing mixing rates, while the activity of aconitase and isocitrate dehydrogenase increases with higher mixing rates. The optimal mixing rate in laboratory bioreactors is 300 to 1000 rpm [57].

Intense mixing also leads to the development of fungal mycelia in short, branched hyphae characterized by high citric acid overproduction. It also contributes to mycelial fragmentation and regrowth. This phenomenon can be beneficial because metabolically inactive and highly vacuolated mycelial fragments are the most susceptible to fragmentation, and this process generates new *Aspergillus niger* mycelial fibers [105].

#### 4.2.7. Temperature

Temperature is another parameter that affects enzymatic activity, microbial transport systems, and consequently, the efficiency of citric acid biosynthesis. In the production of citric acid, the optimal temperature falls within the range of 28 °C to 30 °C. Numerous studies have shown that the highest process efficiency was achieved at a temperature of 30 °C [122,128,129,130,131]. Temperatures above 30 °C lead to the denaturation of citrate synthase, limiting citric acid accumulation and biomass growth in the medium while promoting oxalic acid production. Cultures at lower temperatures decrease enzymatic activity [122,131].

### 4.3. Substrate

The search for citric acid biosynthesis substrates involving *Aspergillus niger* has been the subject of extensive research [54,123,132,133,134,135,136,137,138,139,140,141]. High-purity substrates such as sucrose, glucose, fructose, and maltose can be utilized in citric acid production. Examples of using high-purity raw materials in the citric acid production process involving *Aspergillus niger* are presented in Table 4.

Sucrose is the most favorable carbon source due to its low molecular weight, facilitating transport across microbial cell membranes, and rapid hydrolysis by invertase activated in low-pH environments [101,142]. High-purity substrates allow for the control of the citric acid production process with a yield greater than 70% [26,90]. However, using pure sugar in industrial citric acid production is associated with high costs, as the raw material cost often exceeds the obtained product’s price [143]. Therefore, in industrial citric acid production involving *Aspergillus niger*, cheap and renewable carbon sources such as agro-industrial waste materials are employed [2,94].

Examples of using agro-industrial waste materials in the citric acid production process with *Aspergillus niger* are presented in Table 5 and Table 6. Environmental concerns and the high pollution control costs drive the interest in utilizing waste and industrial effluents in citric acid biosynthesis. Industrial by-products used in citric acid production include beet molasses [144,145,146], sugarcane molasses [147], cellulose [148], lipids [137,149], whey, fruit pomaces [92,150,151], inulin [152], starch materials [121,134,153,154], sweet potatoes [155,156], cassava [4,136], seaweed [122], and glycerol [157,158]. Industrial waste is considered the best carbon source in solid-state cultures [32].

The primary raw materials in citric acid production involving *Aspergillus niger* are cane and beet molasses, primarily due to their low cost [50,93]. Molasses is characterized by a high carbohydrate content, approximately 50%, mainly in sucrose, glucose, and fructose. However, its chemical composition is variable and heterogeneous, which could hinder the biosynthesis process. To enhance the quality of the culture medium, molasses undergoes various treatments involving ferrocyanide, hydrochloric acid, tricalcium phosphate with hydrochloric acid, ammonium oxalate, ammonium dihydrogen phosphate, and lime, as well as fractionation [119,159,160,161].

**Table 4:** High-purity substrates used in citric acid production by *Aspergillus niger* strains.

Substrate	Strain	Substrate Concentration	Cultivation Method	Yield	References
Sucrose	*Aspergillus niger* C–12	150 g∙dm^−3^	SF	77.7% (m/m)	[26]
	*Aspergillus niger* C–12	150 g∙dm^−3^	SF	81.2% (m/m)	
	*Aspergillus niger* NCIM705	60 g∙dm^−3^	SF	30.7 g∙dm^−3^	[162]
Glucose	*Aspergillus niger* PM–1	150 kg∙m^−3^	SF	121 kg∙m^−3^	[86]
	*Aspergillus niger* Yang no. 2.	0.12 mg∙dm^−3^	SF	15.4 mg∙mL^−1^	[112]
Galactose	*Aspergillus niger* ATCC 12846	100 g∙dm^−3^	SF	0.3%	[115]
	*Aspergillus niger* ATCC 26036	100 g∙dm^−3^	SF	0.4%	
	*Aspergillus niger* ATCC 26550	100 g∙dm^−3^	SF	0.1%	
	*Aspergillus niger* IMI 31821	100 g∙dm^−3^	SF	2.3%	
	*Aspergillus niger* IMI 83856	100 g∙dm^−3^	SF	1.5%	
Starch hydrolysates	*Aspergillus niger* UE–1	15% (glucose equivalent)	LSF	490 g∙kg^−1^	[163]
Starch	*Aspergillus niger* GCB–47	150 g∙dm^−3^	SF	45.1 g∙dm^−3^	[164]
	*Aspergillus niger* GCMC	150 g∙dm^−3^	SF	69.5 g∙dm^−3^	
Anhydrous glycerol	*Aspergillus niger* W78B	150 g∙dm^−3^	SF	59.0 g dm^−3^	[165]
Anhydrous glycerol	*Aspergillus niger* PD66	100 g∙dm^−3^	SF	64.2% (m/m)	[166]
Anhydrous glycerol + sucrose	*Aspergillus niger* PD66	135 g∙dm^−3^ +15 g∙dm^−3^	SF	95.80% (m/m)	[167]

**Table 5:** Agricultural and industrial wastes used in citric acid production by *Aspergillus niger* strains.

Substrate	Strain *Aspergillus niger*	Cultivation Method	Yield	References
Sugarcane bagasse	*Aspergillus niger* ATCC 9142	SSF	97.81 g∙kg^−1^	[168]
	*Aspergillus niger* 14/20	SSF	50.01 μg∙g^−1^	[147]
	*Aspergillus niger* DS 1	SSF	31.8%	[169]
Sugarcane molasses	*Aspergillus niger* ATCC 9142	SF	106.65 g dm^−3^	[170]
	*Aspergillus niger* EB–3	SSF	0.112 mg∙dm^−3^	[171]
	*Aspergillus niger* GCMC–7	SF	96.1 g dm^−3^	[161]
Beet molasses	*Aspergillus niger* A20	SLF	29.7 g dm^−3^	[172]
	*Aspergillus niger* A20	SF	8.6 g dm^−3^	
	*Aspergillus niger* W78B	SF	110 g dm^−3^	[173]
Cassava	*Aspergillus niger* FUO–2	SF	88.73 g dm^−3^	[136]
	*Aspergillus niger* NRRL 2001	SSF	88.0 g∙kg^−1^	[36]
Pineapple waste	*Aspergillus niger* DS 1	SSF	54.2%	[174]
Apple waste	*Aspergillus niger* NRRL 567	SSF	65.6	[151]
	*Aspergillus niger* NRRL 567	SF	8.3 g dm^−3^	
Fruit waste– *Parkia biglobosa*	*Aspergillus niger*	SF	1.15 g dm^−3^	[123]
Palm oil	*Aspergillus niger* IBO–103MNB	SSF	337.94 g∙kg^−1^	[149]
Starch	*Aspergillus niger* ATCC 9142	SF	2.7 g dm^−3^	[139]
Whey	*Aspergillus niger* ATCC 9642	SFC	2.43 g dm^−3^	[175]
Date syrup	*Aspergillus niger* J4	SF	56.7 g dm^−3^	[176]
Peat	*Aspergillus niger* NRRL 567	SF	82.0 g∙kg^−1^	[177]
Distillery stillage	*Aspergillus niger* ATCC 9142	SSF	6.15 g∙kg^−1^	[178]
	*Aspergillus niger* ATCC 201122	SF	71.63%	[179]
Molasses (14%) + corn starch (14%) + sucrose (5%)	*Aspergillus niger* NCIM 1055	SF	0.13 mg∙dm^−3^	[121]
Corn starch + sucrose (15%)	*Aspergillus niger*	SSF	138.24 g∙kg^−1^	[134]
Date waste + whey	*Aspergillus niger* ATCC 6275	SF	32.4 g dm^−3^	[180]
Orange waste + cane molasses	*Aspergillus niger* von Tiegh 1867	SF	640 g∙kg^−1^	[181]
Grape waste + sucrose (15%)	*Aspergillus niger*	SSF	34.4 g∙kg^−1^	[182]
Lime waste + sucrose (15%)			28.6 g∙kg^−1^	

Apart from cane and beet molasses, starch-based and lignocellulosic products are considered inexpensive and readily available carbon sources due to their high carbohydrate content [106,121,183,184]. However, these resources are limited due to contamination with heavy metals, amino acids, or proteins and the need for hydrolysis. Citric acid biosynthesis from non-hydrolyzed starch-based materials can be conducted using amylolytic strains of *Aspergillus niger*. This process, however, is characterized by low efficiency [90,106]. For starch-based materials, enzymatic hydrolysis includes treatment with *α*-amylase, amyloglucosidase, isoamylase, or pullulanase [90]. For cellulose hydrolysis, *β*-endoglucanase, *β*-exoglucanase, and *β*-glucosidase are used [183].

There are reports in the literature on using *Aspergillus niger* strains for citric acid biosynthesis using glycerol as the sole carbon source, but this issue has not been widely studied [107,157,158,160,165]. The use of pure glycerol as the primary carbon source was investigated by Foryś et al. Their studies obtained 59.0 g∙dm^−3^ of citric acid produced with a yield of 0.39 g∙g^−1^ [136].

In studies using glycerol as a carbon source in *Aspergillus niger* cultures, it was mainly used with other substrates. Schneider et al. used glycerol as an additive in concentrations ranging from 0 to 40% in solid substrates composed of waste from tung oil production as the primary carbon source. They achieved the highest yield (350.0 g∙kg^−1^) after seven days of cultivation with a 20% glycerol addition [158].

Bauwelers and Grosenker, in surface cultures using molasses as the primary carbon source with a 30% addition of waste glycerol, which had been previously treated with CaO at a concentration of 3.0 g∙kg^−1^, achieved a 95% yield in citric acid biosynthesis. In their submerged cultures, using substrates containing 70% cassava flour, 20% cornmeal, and 10% waste glycerol, they obtained an 88% yield in citric acid biosynthesis. However, in substrates containing 60% cassava flour, 20% cornmeal, and 20% waste glycerol, the process yield was slightly lower, at 85% (Table 6) [157].

**Table 6:** Examples of using glycerol for citric acid biosynthesis by strains of *Aspergillus niger*.

Substrate	Strain *Aspergillus niger*	Cultivation Method	Yield	References
Molasses (70%) + waste glycerol (30%)	*Aspergillus niger*	SLF	95%	[157]
Cassava flour (70%) + corn flour (20%) + waste glycerol (10%)		SF	88%	
Cassava flour (60%) + corn flour (20%) + waste glycerol (20%)		SF	85%	
Glucose (80%) + waste glycerol (20%)		SF	90%	
Waste glycerol	*Aspergillus niger* PD66	SF	6.2% (m/m)	[166]
Waste glycerol	*Aspergillus niger* PD66	SF	114.14 g dm^−3^	[185]

There is limited scientific literature on the biosynthesis of citric acid by *Aspergillus niger* strains on glycerol-based substrates, even though they are considered the best acid producers and are used in industrial production. The lack of interest is likely due to the belief that glycerol slows down the growth rate of filamentous fungi and is not conducive to citric acid production by *Aspergillus niger* [186].

The intensive growth of research into glycerol biotransformation by microorganisms is driven by the challenge of managing the glycerol phase resulting from biodiesel production and the increasing demand for industrially valuable products, such as citric acid, docosahexaenoic acid, propionic acid, lactic acid, and dihydroxyacetone. Glycerol metabolism is of great importance due to the production of double the amount of reducing equivalents compared to glucose metabolism, indicating that glycerol provides more energy for further conversions [187].

In addition to the type of carbon source, its concentration also plays a significant role in the process of citric acid biosynthesis. High efficiency and rapid citric acid biosynthesis are achieved with carbohydrates rapidly taken up and metabolized by filamentous fungi. The effect of carbon source concentration and type on citric acid accumulation depends on the properties of phosphofructokinase-1. Under physiological conditions, its activity is inhibited by citric acid at concentrations of 1–5 mM. However, this inhibition of enzyme activity does not occur during fermentation. This is because high concentrations of sucrose or glucose are used, which leads to an increase in fructose-2,6-bisphosphate, a potent activator of phosphofructokinase-1. High carbohydrate concentrations also induce premeases, allowing for the rapid uptake of carbon and, as a result, glycolysis. Also, high carbon source concentrations significantly regulate pyruvate carboxylase activity [49,188,189]. Carbohydrates in concentrations exceeding 200.0 g∙dm^−3^ lead to a reduction in the rate of citric acid biosynthesis. This reduction may be due to an increase in fungal biomass concentration, elevated medium viscosity, and the synthesis of polyalcohols. On the other hand, carbohydrate concentrations below 50.0 g∙dm^−3^ result in low citric acid biosynthesis efficiency and the accumulation of oxalic acid [50,190,191].

## 5. Application of Citric Acid in the Food Industry

From a health quality perspective, citric acid, when used as a food additive, has been approved as generally recognized as safe (GRAS) by the FAO/WHO Expert Committee on Food Additives, and its Acceptable Daily Intake (ADI) does not require limitation [54]. Derivatives of citric acid, such as calcium citrate, iron citrate, manganese citrate, potassium citrate, sodium citrate, diammonium citrate, isopropyl citrate, and stearyl citrate, have also received GRAS status as food additives [13].

Citric acid is characterized by its low production cost, easy accessibility, non-toxicity, biocompatibility, universality, and the safety of its decomposition products. It finds wide applications in the food, pharmaceutical, biomedical, chemical, agricultural, and environmental protection industries [92]. Examples of citric acid applications in the food industry and other sectors are presented in Table 7 and Table 8. In food products, citric acid serves various functions, including acidity regulation, preservation, antioxidant properties, emulsification, flavor and aroma enhancement, buffering, and antibacterial activity. Citric acid’s ability to chelate metal ions and its buffering properties, when combined with citrates, make it an ideal additive in food and nutraceutical production [42,192,193].

Citric acid plays a significant role as an antioxidant in oil production and in limiting the oxidation of lipids in meat processing. It inhibits lipid oxidation by forming bonds between pro-oxidative metal ions and the carboxyl or hydroxyl groups of the acid. The antioxidant activity of citric acid in food depends on the dose applied and increases with higher acid concentrations [194].

In meat processing, citric acid reduces the pink color and increases the brightness of heat-treated meat. In cooked meat, citric acid limits the endogenous pink color and the color induced by sodium nitrite and nicotinamide. Reduction of the pink color in meat may also result from the chelation of heme iron by citric acid, preventing heme from binding with ligands that cause a pink color [195,196].

**Table 7:** Examples of citric acid applications in the food industry.

Industry	Application	References
Beverages—wines, juices, non-alcoholic beverages, syrups	Used as an acidity regulator in carbonated and non-carbonated beverages, a buffering agent, pH regulator	[12,197]
Sweets—jams, jellies, candies	Used as an antioxidant, antibacterial agent, controlling sugar inversion and product pH for optimal gelling, preservative, providing a bitter taste and enhancing flavor	[192]
Dairy products	Sodium citrate is used in cream production to stabilize casein, prevent the formation of creams during hot milk beverage production, and act as an emulsifier to stabilize the water and oil phases in cheese production. Aqueous solutions of citric acid are used for milkstone removal from equipment	[14]
Meat products	It acts as a chelating agent, helping maintain the natural color and prevent discoloration of preserved meats; acts as an antioxidant and synergist for antibacterial agents; Sodium citrate is used in slaughterhouses to prevent coagulation or clotting of fresh blood	[32]
Fruit and vegetable industry	Citric acid, along with ascorbic acid, inhibits enzyme activity and oxidation reactions that may deteriorate colors and flavors	[192]
Oils	Used in the deodorization and hydrogenation of oil to chelate metal ions, catalyze the rancidity of fats, interrupt the formation of peroxides and other oxidation products in the auto-oxidation of oils	[37]
Seafood	Prevents discoloration and the development of unwanted odors by chelating metals	[12]

Citric acid and its salts are widely used in the food industry to prevent enzymatic browning [198]. Enzymatic browning of fruits and vegetables is a phenomenon that reduces shelf life and influences consumer decisions [199,200].

Citric acid is used as an additive in rinse water before deep freezing and in fruit syrups [201]. Adding citric acid to stored fruits and vegetables positively affects color retention and organoleptic quality and extends their shelf life. It can also be combined with other anti-browning agents, such as ascorbic acid [202].

The inhibition of citric acid’s polyphenol oxidase (PPO) activity is due to its pH-lowering capacity. PPO activity gradually decreases with increasing citric acid concentrations. However, the citric acid concentration needed to inhibit PPO activity varies depending on the PPO activity and buffer solutions used [198]. Radish slices immersed in a 0.3% aqueous citric acid solution showed no browning during storage. Querioz et al. found that a citric acid concentration of 100 mM inhibited the PPO activity of cashew apples [199]. However, a 10 mM citric acid solution inhibited banana PPO [148].

Citric acid also contributes to a decrease in the thermodynamic parameters of polyphenol oxidase. This is believed to be due to a reduction in the stability of PPO and the number of non-covalent bonds in the enzyme’s structure, leading to changes in the protein’s secondary and tertiary structure [198].

Citric acid can be considered a substance capable of controlling cellular respiration and contributing to the better preservation of fruits and vegetables during storage [200,203].

**Table 8:** Examples of citric acid applications in various industries.

Industry		Application	References
Pharmaceutical industry	Medicines, pharmaceutical preparations, blood banks	It is used as an anticoagulant, effervescent in combination with bicarbonates or carbonates, a flavoring agent, and a stabilizer. It imparts the desired sour taste, which helps mask medicinal flavors	[8,14,204,205]
Cosmetics industry	Detergents, cosmetics	It is added to hair care products, cosmetics, and detergents for pH regulation and used as a stabilizer, buffering agent, and chelating agent to prevent discoloration	[14,206]
Agriculture	Animal feeds	Enhances the bioavailability of mineral nutrient chelates, improves taste, regulates stomach pH, and enhances the effectiveness of animal feeds; used as a flavor enhancer in pet food	[207]
	Fertilizers	Forms chelate with Fe, Cu, Mg, and Zn, used for soil correction, increase phosphorus availability to plants, are employed to remove lead from contaminated soils, and are used for copper chelation in algaecides for water reservoirs	[208]
Other applications in industry	Textile industry	It is used for pH regulation, as a buffer, and as a chelating agent in the dyeing process	[37]
	Metallurgical industry	Cleans steam boiler from metal oxides and purifies iron and copper oxides used in nuclear reactor welding	
	Electroplating	It is used as a chelating agent to control the metal deposition rate on substrates	
	Biomedical engineering	Utilized as a copolymer in nanomaterials to encapsulate biologically active compounds	[209]
	Water purification	Solutions of citric acid are used to remove iron, calcium, and other cations that damage cellulose acetate membranes used in reverse osmosis systems	[210]

### 5.1. Newly Emerging Applications of Citric Acid

Research into new applications of citric acid in various industries is currently the subject of many studies [211,212,213,214,215,216]. One of the new applications of citric acid is the production of household detergents. Citric acid chelates Mg^2+^ and Ca^2+^ ions responsible for water hardness and does not contribute to the eutrophication of aquatic systems, unlike phosphates used in detergents [217]. New and innovative applications of citric acid in the food industry and beyond are expected to lead to increased production.

#### 5.1.1. Cross-linking Agent and Plasticizer

Citric acid can successfully be used in the process of cross-linking proteins [218], polysaccharides [219], and hydroxyapatite [220]. A breakthrough in using citric acid as a cross-linking agent came with the discovery by Rothenberg and Alberts from the University of Amsterdam. They demonstrated that glycerol and citric acid can polymerize, creating a water-soluble, biodegradable, and thermosetting resin. The combination of citric acid and glycerol at temperatures above 100 °C and below 130 °C under normal conditions leads to the formation of polyester resins through the Fischer esterification reaction [221].

The use of citric acid as a compatibilizer for various polysaccharides, including starch, thermoplastic starch, cotton, chitosan, and cellulose, is justified by its multi-carboxyl structure [222]. This allows it to be used as a cross-linking agent, plasticizer, and hydrolyzing agent [223].

The mechanism of the cross-linking reaction is based on the well-known Fischer esterification reaction between the carboxyl groups of citric acid and the hydroxyl groups of starch [224]. Citric acid can react with all three hydroxyl groups of starch. Esterification between starch and citric acid leads to mono-, di-, and tri-esters forming. Esterification primarily occurs within the branching points of amylopectin [225]. The formation of ester bonds can be catalyzed by reducing the pH or adding Lewis acids, which are chemical compounds capable of accepting an electron pair from a base [224]. In many previous studies on starch cross-linking, temperatures above 100 °C have initiated the cross-linking process [226]. Heating citric acid causes dehydration and the formation of an anhydride, which can react with starch to form starch citrate. With further heating, the citrate undergoes dehydration, and cross-linking can occur [227,228].

It is possible to conduct cross-linking reactions at lower temperatures (around 70 °C) using higher concentrations of citric acid. However, the efficiency of the reaction under these conditions is low because only a tiny amount of added citric acid participates in the cross-linking reaction. Furthermore, citric acid that has not reacted can act as a plasticizer [225]. As a plasticizer, citric acid increases the tensile strength of starch films. The improvement in tensile strength is more significant than when glycerol is used as a plasticizer [229]. Excessive citric acid concentration does not interact with starch molecules but can react with water, disrupting hydrogen bonds and reducing the matrix’s cohesion. This results in increased water solubility, susceptibility to deformation, and reduced thermal resistance [153].

FTIR spectroscopy and X-ray diffraction of starch films with citric acid have shown that citric acid can effectively inhibit starch recrystallization or retrogradation due to strong hydrogen bonding between starch and citric acid [224]. Additionally, citric acid-cross-linked starch films exhibit significantly higher tensile strength, up to 150% more than non-cross-linked films. However, achieving the appropriate increase in material strength requires an optimal amount of citric acid [230]. Citric acid concentrations below 5% act as a cross-linking agent and enhance the tensile strength of starch films. When the concentration increases from 5% to 30%, tensile strength decreases, but flexibility and material adhesiveness increase. This suggests that excess free citric acid is a plasticizer [231].

The main disadvantage of citric acid as a cross-linking and plasticizing agent in starch barrier films is starch degradation due to acid hydrolysis. Acid hydrolysis of starch glycosidic bonds involves the cleavage of these bonds, resulting in the protonation of oxygen and the addition of a water molecule, leading to the formation of a reducing sugar group. Effectively preventing starch hydrolysis during cross-linking in the presence of citric acid can be achieved by maintaining a pH of 4 or lower and a temperature below 105 °C [224].

As a cross-linking agent, citric acid strengthens bonds by incorporating covalent bonds that complement intermolecular hydrogen bonds, improving the resistance of starch films to moisture. Strong hydrogen bonds between the carboxyl groups of citric acid and the hydroxyl groups of starch result in improved interactions between molecules and reduced solubility of the films in water [153].

When citric acid is added in the range of 1% to 10% to thermoplastic starch, it significantly reduces water vapor permeability. This effect is due to replacing hydrophilic groups with hydrophobic ester groups that impede the diffusion of water vapor molecules through the matrix. However, water vapor permeability increases when the citric acid concentration exceeds 10%. This can be attributed to the plasticizing effect caused by an excess of citric acid. Increased citric acid concentration leads to enhanced chain mobility and increased interchain spaces, resulting from the attachment of free citric acid to the polymer chain. As a result, the water vapor diffusion coefficient increases, accelerating water vapor penetration through the starch films [232,233,234].

Starch films cross-linked with citric acid have higher thermal resistance [226]. The reason for this is that the cross-links are responsible for the resistance of cross-linked films. Cross-linked starch films exhibit significantly improved thermal resistance at temperatures above 320 °C [230].

The three carboxyl groups and one hydroxyl group in citric acid also allow it to cross-link glycerol, cellulose, and sebacic acid through condensation reactions, forming ester copolymers capable of drug delivery [8]. Gentamicin incorporated into the polymer effectively kills bacteria. Citric acid delivers ketoconazole as a cross-linking agent for beta-cyclodextrins on hydrogel hydroxypropylmethylcellulose (HPMC) membranes. The formation of drug-cyclodextrin complexes contributes to increased solubility and bioavailability of poorly soluble drugs [235].

#### 5.1.2. Citric Acid in the Synthesis of Deep Eutectic Solvents

Deep eutectic solvents (DES) are homogeneous mixtures of two or more components capable of interacting with each other as a hydrogen bond acceptor (HBA) and a hydrogen bond donor (HBD). Deep eutectic solvent mixtures are formed by mixing two or more components in the appropriate molar ratio in the presence of heat. Additionally, this process does not require an additional purification step [236,237]. Deep eutectic solvents are one of the most promising discoveries in “green chemistry”. They can serve as an alternative to conventional organic solvents and have numerous advantages, such as renewability, reusability, biodegradability, non-toxicity, widespread availability, shallow vapor pressure, low flammability, and ease of preparation. Moreover, the components that produce deep eutectic solvents are inexpensive and safe [238,239].

Deep eutectic solvents have been divided into four types depending on their composition. Types I, II, and IV contain metal salts and are considered toxic and less sustainable than type III deep eutectic solvents. Type III deep eutectic solvents are synthesized from readily biodegradable and regenerable raw materials such as feed additive (choline chloride, ChCl), fertilizer (urea), antifreeze (ethylene glycol), sweetener (glycerol), and plant metabolites (sugars, sugar alcohols, and organic acids) [240]. The most frequently studied eutectics in the literature are type III, based on the combination of quaternary ammonium salts and a compound serving as a hydrogen bond donor. Type III is the most commonly used deep eutectic solvent due to the strong interaction of hydrogen bonds between the hydrogen bond acceptor (HBA) and the hydrogen bond donor (HBD). Many compounds have been successfully utilized to create deep eutectic solvents. HBAs are mainly quaternary ammonium or phosphonium salts, while HBDs are most commonly amides, alcohols, and carboxylic acids. Citric acid is one of the most commonly employed HBDs among carboxylic acids [237]. The most popular systems for producing deep eutectic solvents using choline chloride and citric acid are 1:1, 1:2, and 2:1 [241,242]. The presence of hydroxyl and carboxyl groups allows for the formation of sufficiently strong hydrogen bonds. Deep eutectic solvents based on choline chloride and carboxylic acids demonstrate greater extraction efficiency than traditional solvents such as water and ethanol [243].

Various molar ratios of choline chloride and citric acid monohydrate significantly influence the physicochemical properties of deep eutectic solvents. Adding citric acid monohydrate increases viscosity, surface tension, and density. Deep eutectic solvents with a higher molar ratio of choline chloride exhibit a higher melting point. Citric acid-based deep eutectic solvents can find broad industrial applications, particularly in extracting hydrophilic components from plant or animal materials [243].

In the studies conducted by Kurtulbaş et al., deep eutectic solvents were intentionally designed, incorporating a hydrogen bond donor (HBD) (glycerol and ethylene glycol) and a hydrogen bond acceptor (HBA) (citric acid) in a specified molar ratio (1:4) for the extraction of bioactive compounds (phenols and anthocyanins). In the current investigation, *Hibiscus sabdariffa* was extracted using microwave-assisted extraction (MAEX). The most effective extract from *Hibiscus sabdariffa* was obtained from a mixture of citric acid and ethylene glycol through microwave-assisted extraction [239].

Hu et al., investigated the molecular mechanisms of isoliquiritigenin extraction using deep eutectic solvents of choline chloride and citric acid. The results indicated that deep eutectic solvents exhibited higher efficiency in isoliquiritigenin extraction as an extraction solvent than ethanol with water. Additionally, the increased efficiency in isoliquiritigenin extraction was primarily attributed to the strong interaction between isoliquiritigenin and the extraction solvent and the rapid diffusion of isoliquiritigenin [237].

#### 5.1.3. Antibacterial Agent

Using organic acids to control bacterial flora in food, extend shelf life, and improve the safety of plant- and animal-derived products has become a common practice in the food industry. In Europe, the legal basis for using organic acids as agents contributing to the safety of animal-derived products is Regulation 853/2004 of the European Parliament and the Council [244,245,246].

**Figure 12:**
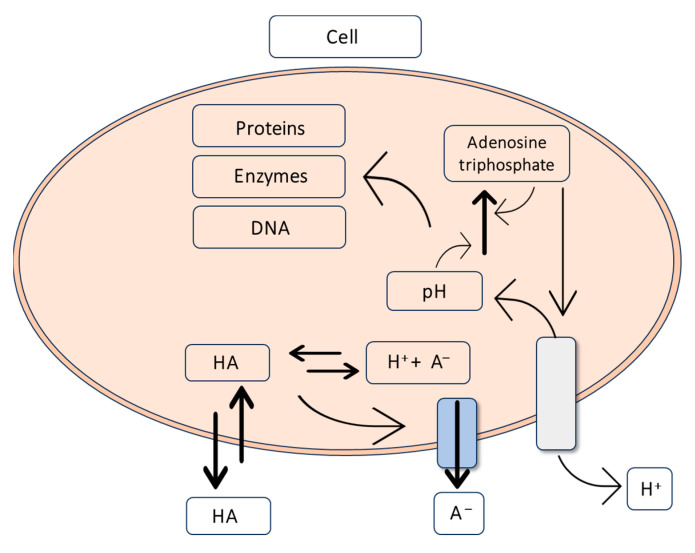
The mechanism of citric acid’s action on a bacterial cell [253].

Citric acid effectively combats pathogenic microflora in fresh and processed pork, beef, poultry, and fresh vegetables and fruits [247,248,249,250,251]. Its antibacterial activity involves penetrating through the cell membranes, where the pH is higher than in the surrounding environment. The mechanism of citric acid’s antibacterial action is related to acidifying the cytoplasm, disrupting metabolic processes, or accumulating the dissociated acid anion to a toxic level (Figure 12) [252,253]. Organic acids are weak acids, so they do not completely dissociate in an aqueous environment, and their microbiological activity depends on the degree of dissociation and the pH of the food product. Reducing the pH increases the concentration of the acid, reduces the polarity of the molecules, improves acid diffusion through microbial cell membranes into the cells, and consequently increases antibacterial activity [253,254]. The effectiveness of organic acids also depends on the acid concentration, acid properties, temperature, exposure time, and microbial susceptibility [251,255].

Citric acid in concentrations ranging from 0.1 to 3.0% restricts the growth of bacteria such as *Listeria monocytogenes*, *Escherichia coli*, *Salmonella typhimurium*, and *Vibrio parahaemoliticus* [244,247,249,251].

Citric acid exhibits synergistic effects when used in mixtures with other organic acids. A mixture of caprylic and citric acids significantly inhibits the growth of bacteria. The synergy between citric and caprylic acids is associated with the loss of cell membrane integrity and changes in its permeability. The mechanism of the synergistic action of both acids involves damaging or destabilizing the cell membrane, leading to increased permeability and, consequently, cell death. Damage to the bacterial membrane allows hydrogen ions to penetrate, resulting in a strong bactericidal effect [248,256,257].

Combining citric acid with other decontamination methods, such as ozonation, UV-C radiation, and ultrasonication, can significantly impact the inactivation of microorganisms in fresh food [41].

The effectiveness of citric acid’s antibacterial action varies and depends on many factors. Citric acid exhibits optimal antibacterial effects in a low pH environment, at low temperatures, and when used in high concentrations. Available literature sources report that using citric acid at concentrations above 2% may cause adverse sensory changes in food products [253,258]. The antibacterial effectiveness of citric acid also depends on the initial amount of microflora on the product’s surface. Depending on the initial bacterial count, citric acid reduces the number of microorganisms by 1 to 2 log cfu∙g^−1^ [244].

#### 5.1.4. Deamidation of Gluten

Wheat gluten is widely used in the food industry, serving various purposes, such as emulsifiers and imparting cohesiveness and elasticity. However, its utility is limited due to its low solubility under neutral conditions. A practical method to enhance the properties of gluten is deamidation using carboxylic acids, including citric acid [259,260].

The deamidation reaction transforms amidic groups into carboxylic groups, mainly glutamine and asparagine residues. This transformation results in increased electrostatic repulsion, the disruption of hydrogen bonds, and the dissociation of polymers, ultimately improving the solubility of gluten. While treating gluten with citric acid, the availability of peptide bonds and hydrogen ions influences the competition between hydrolysis and deamidation. The degree of hydrolysis of deamidated gluten decreases with increased citric acid concentration and treatment time, contributing to an increase in soluble protein content after deamidation [215,261]. Deamidation by citric acid increases the solubility of gluten to around 70% at pH 7. The shift of the protein’s isoelectric point towards acidic pH confirms that deamidation increases the quantity of protein polyelectrolytes, resulting in improved solubility at neutral pH. Deamidation involves the cleavage of peptide bonds, indicating that it is primarily responsible for increasing protein solubility rather than hydrolysis [261,262].

Deamidation of gluten with citric acid significantly enhances its emulsifying, foaming, and elasticity properties. Emulsions stabilized with deamidated gluten feature smaller emulsion droplet sizes, indicating their ability to reduce surface tension. High emulsion stability results from the increased flexibility of the gliadin molecule or its molecular rearrangement [261]. The improvement in the foaming capacity of deamidated gluten is attributed to the increased molecule flexibility, leading to enhanced protein adsorption and anchoring at phase boundaries [263,264]. Deamidation also leads to changes in the secondary conformation of the protein, driven by increased electrostatic repulsion and a reduction in hydrogen bonds [262]. The secondary structure of gluten consists of 34.5% α-helices, 17.3% *β*-turns, and 44.8% *β*-sheets. Deamidation increases *α*-helices and *β*-turns while reducing *β*-sheets [260,263].

Citric acid exhibits a solid capability to break peptide bonds in gluten, which leads to a transformation in the tertiary structure of the protein. Protein fractions of gluten with a higher molecular weight are more susceptible to degradation than those with a lower molecular weight. After deamidation with citric acid, the presence of sulfhydryl groups in gluten has been confirmed, while the tertiary structure becomes less compact [263,264].

Deamidation with citric acid can contribute to the improvement of the nutritional properties of gluten. From a nutritional standpoint, gluten is not considered a good protein source due to its deficiency in lysine and threonine. Deamidation with citric acid increases the overall quantity of essential amino acids, including lysine [261].

#### 5.1.5. Extractant

Citric acid can be an effective pectin extractor from fruit pomace instead of toxic mineral acids such as sulfuric or nitric acid. Pectin extraction using citric acid can reduce waste from fruit and vegetable processing and limit the harmful environmental impact of wastewater from conventional extraction methods [265,266].

The process of pectin extraction typically occurs at around 97 °C with a pH of 2.5 using water acidified with citric acid [193,265].

Extracting pectin from citrus fruit peels using citric acid allows for obtaining pectins with a high galacturonic acid content, essential for their application as a gelling agent. Pectins also have a high molecular weight, indicating a high content of neutral sugars. The viscosity of the extracted pectins increases with a higher concentration of citric acid. Although the extraction process may yield lower efficiency than traditional methods, it results in pectins of high purity [267].

The pectins extracted from cocoa husks were characterized by a high degree of acetylation, contained rhamnogalacturonan, and had side chains rich in galactose. Despite their high degree of acetylation, these pectins formed gels in a low pH environment and at a high glucose concentration, suggesting their potential use as an additive in acidic products [268].

#### 5.1.6. Inhibition of Protein Adhesion

The issue of protein adhesion to steel surfaces poses numerous challenges in food production and processing. Proteins adhering to equipment surfaces can serve as a nutrient source for microorganisms, leading to product contamination. Removing allergens and preventing cross-contamination is a critical point in the food production process. One method to inhibit the adhesion of chicken egg white proteins to stainless steel surfaces is to use a citric acid solution [269].

The effect of inhibiting protein adhesion by citric acid involves changing the surface charge of steel from positive to negative in an environment with a pH of 7.4 due to the attachment of dissociated carboxyl groups from the acid. This leads to the repulsion of negatively charged protein molecules such as ovalbumin or ovomucoid [216,269].

## 6. Global Citric Acid Market

Acidity regulators are a significant part of the food additives industry, as they impact the taste of food and contribute to its shelf life. The acidity regulators market is estimated to reach USD 7.29 billion in 2023, with a CAGR of 7.09%. Citric acid dominates the acidity regulators market due to its wide applications in the non-alcoholic beverage industry and the increasing societal preference for safe food [270]. In 2016, approximately 67% of the citric acid produced worldwide was used in the food industry, 16% in the chemical industry, 8% in pharmaceuticals and cosmetics, and 7% in other sectors such as textiles and metallurgy [271].

In the 1930s, American companies Miles and Pfizer led citric acid producers with 4900 tons [27]. However, by 1978, the combined production of Pfizer and Miles had reached approximately 70,000 tons. In 1990, the annual production of citric acid had already reached 170,000 tons. Even though the largest citric acid producers in the 1990s were located in the United States, Europe produced around 255,000 tons annually. At that time, North America had 215,000 tons, Asia produced 66,000 tons, Africa produced 14,000 tons, Australia produced 8000 tons, and South America produced 7000 tons of citric acid per year [272].

**Figure 13:**
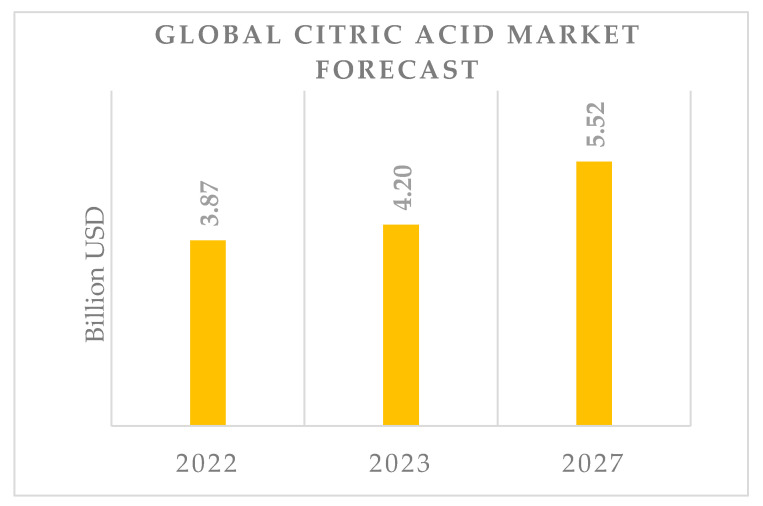
Global citric acid market forecast.

The current citric acid market is considered the fastest-growing segment in the food additives industry, driven by its wide range of applications in various industries and the increasing use of citric acid as a cleaning agent. The global citric acid market increased from USD 3.87 billion in 2022 to USD 4.2 billion in 2023. The Russia-Ukraine war has ruined, at least in the short term, the chances of reviving the global economy after the COVID-19 pandemic. The war between the two countries has led to economic sanctions on many countries, rising commodity prices, and supply chain disruptions, causing inflation in goods and services and affecting many markets worldwide. The citric acid market is expected to grow to USD 5.52 billion in 2027 (Figure 13) [95]. The global citric acid market reached a volume of 2.8 million tons in 2022, and forecasts suggest that by 2028, the market will reach 3.3 million tons, indicating a CAGR of 2.78% for 2022–2028 [270].

The global citric acid market is divided among North America, Latin America, Western Europe, Eastern Europe, and Asia-Pacific, excluding Japan (APEJ). In 2016, the APEJ region dominated the citric acid market in regards to value. Regarding market size, Western Europe (including the United Kingdom, Germany, Italy, France, and Spain) led with production exceeding 500,000 tons. Following it were the APEJ region, North America (including the United States, Canada, and Mexico), and the rest of the world. The global COVID-19 pandemic significantly impacted the citric acid market in 2020. This resulted in a sharp increase in the sales of detergents and cleaning products and a substantial increase in the demand for citric acid [270]. The citric acid market is experiencing significant growth in Western Europe, driven by the established food and beverage sector, where citric acid is widely used as a preservative and flavor enhancer. The growing trend toward natural and organic products in countries such as Germany, France, and the UK creates opportunities for citric acid. Furthermore, the pharmaceutical sector in Western Europe is highly advanced and rigorously regulated, creating demand for trusted and high-quality ingredients, including citric acid, for use in medicines and dietary supplements [3]. On a global scale, the Asia-Pacific region is the largest consumer of citric acid, accounting for 28%. North American countries, with a consumption rate of 23%, come second, while Western Europe, with a share of 22%, ranks third [271].

Key players in the citric acid market include Archer Daniels Midland Company (Chicago, IL, USA), Cargill Inc. (Shanghai, China), Tate & Lyle PLC (London, UK), Jungbunzlauer Suisse AG (Des Plaines, IL, USA), Cofco Biochemical (Anhui) Co., Ltd. (Bengbu, China), Huangshi Xinghua Biochemical Co. Ltd. (Huangshi, China), RZBC Group Co. Ltd. (Rizhao, China), Weifang Ensign Industry Co., Ltd. (Weifang, China), Gadot Biochemical Industries Ltd. (Haifa, Israel), S.A. Citrique Belge N.V. (Tienen, Belgium) [273]. Manufacturers focus on diversifying and delivering high-quality products that meet customer requirements to maintain a competitive edge. Furthermore, companies compete by investing in developing next-generation products with high solubility and acceptable taste. An example is Gadot Biochemical Industries, which introduced a new product called Cal_2_Mg in 2022, a combination of calcium and magnesium citrate. In the same year, Jungbunzlauer introduced a new product called monomagnesium citrate, a one-to-one molar ratio magnesium salt mainly used as a mineral source in functional foods, beverages, and dietary supplements [274].

Currently, a challenge in the citric acid market is the pressure from imports originating in Asian countries, which has decreased selling prices. Additionally, European producers contend with high production costs. In 2021, the European Commission reaffirmed decisions made in 2016 and re-imposed anti-dumping duties on importing citric acid from the People’s Republic of China and Malaysia [275].

## 7. Conclusions

This literature review discusses the properties of citric acid, cultivation conditions primarily for *Aspergillus niger*, differences between filamentous fungi *Aspergillus niger* and yeast *Yarrowia lipolytica*, extensive applications of citric acid in the industry, and the global market for citric acid. The review indicates a growing interest in the microbiological production of citric acid and technological advancements in this area, aligned with market demands.

Additionally, the article emphasizes the use of waste substrates, reflecting the development of a closed-loop economy model. Such a model can reduce economic and environmental costs for producing enterprises. The aspect of exploring new, more cost-effective substrates from the agri-food industry could contribute to cost reduction and solutions for waste disposal issues.

A multifaceted view of citric acid arises from its wide-ranging applications in various industrial sectors and the exploration of new uses. The continuous increase in demand for citric acid is associated with the most economically viable industrial production process, characterized by high sensitivity, complexity, and dependence on parameters such as microorganisms used in substrates and fermentation techniques. In the bioproduction of citric acid, there is significant interest in improving process efficiency at relatively low costs. This serves as an incentive for the development of new technologies and innovations in the design of bioreactors, process scaling, and control. Metabolic engineering can also improve fermentation parameters, requiring substantial research efforts.

The review highlights the importance of *Aspergillus niger* as a versatile industrial microorganism, which, in biotechnological studies, will undergo further investigations into secondary metabolism, fermentation conditions in citric acid production, and enhancing efficiency through CRISPR-Cas9 genome editing.

## Data Availability

Not applicable.
